# Association of Cataract Surgery With Risk of Diabetic Retinopathy Among Asian Participants in the Singapore Epidemiology of Eye Diseases Study

**DOI:** 10.1001/jamanetworkopen.2020.8035

**Published:** 2020-06-16

**Authors:** Yih-Chung Tham, Lei Liu, Tyler Hyungtaek Rim, Liang Zhang, Shivani Majithia, Miao Li Chee, Nicholas Y. Q. Tan, Kah-Hie Wong, Daniel Shu Wei Ting, Charumathi Sabanayagam, Jie Jin Wang, Paul Mitchell, Tien Yin Wong, Ching-Yu Cheng

**Affiliations:** 1 Singapore Eye Research Institute, Singapore National Eye Centre, Singapore; 2 Duke-NUS Medical School, Singapore; 3 Department of Ophthalmology, The First Affiliated Hospital of China Medical University, Shenyang, China; 4 Yong Loo Lin School of Medicine, National University Health System, Department of Ophthalmology, National University of Singapore, Singapore; 5 Centre for Vision Research, The Westmead Institute for Medical Research, Westmead Hospital, Department of Ophthalmology, The University of Sydney, Westmead, New South Wales, Australia

## Abstract

**Importance:**

Cataracts and diabetic retinopathy (DR) are the leading causes of acquired blindness worldwide. Although extraction is the standard treatment option for cataracts, it is also reported to increase the risk of developing DR among individuals with diabetes. Nevertheless, the association between cataract surgery and risk of DR is still not well understood, and there have been no prior population-based reports in this area.

**Objective:**

To assess the risk of developing DR after cataract surgery among individuals with type 2 diabetes.

**Design, Setting, and Participants:**

A population-based prospective cohort study was conducted among participants recruited from the Singapore Epidemiology of Eye Diseases Study. The baseline visit was conducted between June 1, 2004, and March 31, 2009, and the 6-year follow-up visit was conducted between June 1, 2011, and July 31, 2016. Statistical analysis was performed from October 1 to 31, 2019.

**Exposures:**

Cataract surgery performed before a follow-up visit, determined based on slitlamp evaluation of lens status at baseline and follow-up visits.

**Main Outcomes and Measures:**

Eyes with incidence of DR were defined as those with the presence of any DR (level ≥15 based on the modified Airlie House classification system, graded from retinal photographs) at 6-year follow-up with no DR at baseline. The association between cataract surgery and incidence of DR was evaluated using a multivariable Poisson regression model with a generalized estimating equation to account for correlation between both eyes.

**Results:**

A total of 1734 eyes from 972 participants with diabetes (392 Malay individuals and 580 Indian individuals; 495 men; mean [SD] age, 58.7 [9.1] years) were included in the analysis. A total of 163 study eyes had already undergone cataract surgery at baseline, and a total of 187 eyes (originally phakic at baseline) underwent cataract surgery any time during the follow-up period. Of these 350 eyes, 77 (22.0%) developed DR. Among the 1384 eyes that never underwent cataract surgery, 195 (14.1%) developed DR. After adjustments for age, sex, race/ethnicity, baseline hemoglobin A_1c_ level, duration of diabetes, random blood glucose level, antidiabetic medication use, hypertension, body mass index, and smoking status, multivariable regression analysis showed that any prior cataract surgery was associated with incidence of DR (relative risk, 1.70; 95% CI, 1.26-2.30; *P* = .001). Subgroup analyses by race/ethnicity showed similar associations in both Malay individuals (relative risk, 1.73; 95% CI, 1.13-2.69; *P* = .02) and Indian individuals (relative risk, 1.93; 95% CI, 1.33-2.80; *P* < .001).

**Conclusions and Relevance:**

The findings of this population-based cohort study suggest that prior cataract surgery was associated with a higher risk of developing DR among individuals with diabetes. Further validation is warranted to confirm this association.

## Key Points

**Question:**

Is cataract surgery associated with an increased risk of diabetic retinopathy?

**Findings:**

In this population-based cohort study of 972 participants with type 2 diabetes (1734 eyes), any prior cataract surgery was significantly associated with a higher risk of diabetic retinopathy after adjustment for confounding factors. Similar associations were observed in both Malay and Indian participants.

**Meaning:**

These findings suggest that patients with diabetes may benefit from more frequent diabetic retinopathy screenings after cataract surgery.

## Teaser

This population-based cohort study assesses the risk of developing diabetic retinopathy after cataract surgery among individuals with diabetes in Singapore.

## Introduction

Cataracts and diabetic retinopathy (DR) are the leading causes of acquired blindness worldwide,^1,2^ which is further compounded by the fact that patients with diabetes have an increased risk of developing cataracts.^3,4^ Thus, many patients with diabetes concomitantly have cataracts and DR. Although extraction is the standard treatment option for cataracts, it is also reported to worsen existing cases of DR, a microvascular complication of diabetes, leading to further vision loss.^5,6,7^ It was postulated that cataract extraction may possibly lead to a breakdown of the blood-retinal barrier and the blood-aqueous barrier and enhanced intraocular inflammatory response, all of which may result in the occurrence of DR in patients with diabetes.^8,9^ For this reason, current DR care guidelines also recommend measures of preoperative stabilization for patients with diabetes and existing vision-threatening DR who undergo cataract extraction.^10^ These measures include optimizing glycemic control and performing panretinal photocoagulation preoperatively to better prevent postsurgery complications such as neovascularization and vitreous hemorrhage among this group of patients.^10^

Nevertheless, the association between cataract surgery and the risk of DR is currently still not well understood, especially among individuals with diabetes, mainly owing to the limited reports in this area. Hong et al^11^ previously reported that pseudophakic eyes (ie, eyes with cataract surgery performed and with an intraocular lens implant inserted) among patients with diabetes were 2.65 times more likely to develop DR in 12 months compared with phakic eyes (ie, eyes with the natural crystalline lens intact). In addition, cataract surgery was also found to be associated with an increased risk of diabetic macular edema postoperatively.^12,13,14^ Conversely, other studies reported no significant difference in incidence rates of DR or diabetic macular edema between patients who had undergone cataract surgery and those who had not.^15,16^ These previous studies were of short follow-up periods of 6 to 12 months after cataract surgery.

To our knowledge, there have been no population-based studies that examined the association of cataract surgery and risk of DR. Taken together, there is currently still a lack of evidence on the association between prior cataract surgery and risk of DR, especially for long-term follow-up and in an Asian population. Hence, we aimed to assess the risk of developing DR after cataract surgery among individuals with diabetes, using 2 Asian population-based cohorts in Singapore.

## Methods

### Study Population

Participants were recruited from the Singapore Malay Eye Study (SIMES) and the Singapore Indian Eye Study (SINDI), which are part of the Singapore Epidemiology of Eye Diseases study series. Details of the study design and methods of SIMES and SINDI have been described in detail previously.^17,18,19^ In brief, an age-stratified random sampling procedure was conducted to select participants from these cohorts. Of these, 4168 Malay and 4497 Indian individuals were eligible for the baseline studies. At baseline visits, 3280 Malay individuals (response rate, 78.7%) were examined from June 1, 2004, to December 31, 2006, and 3400 Indian individuals (response rate, 75.6%) were examined from January 1, 2007, to March 31, 2009. Of these, 2372 individuals had type 2 diabetes at baseline. Among these individuals, 1844 had diabetes at baseline but did not have DR. After excluding 209 baseline participants who died, 91 with terminal illness and 52 who had moved or migrated, the remaining 1492 eligible individuals were invited to participate in the 6-year follow-up studies of SIMES-2 (June 1, 2011, to December 31, 2013) and SINDI-2 (January 1, 2014, to July 31, 2016) (Figure). Of these, 397 individuals did not return, leaving 1095 participants who attended the 6-year follow-up studies (with a follow-up rate among eligible individuals of 73.4%). Last, 123 participants (261 study eyes) were further excluded owing to ungradable retinal images or missing data of relevant covariates, thus leaving 972 participants (392 Malay individuals and 580 Indian individuals) and 1734 study eyes (690 Malay eyes and 1044 Indian eyes) included in the final analysis (Figure). Both baseline and follow-up studies were conducted in accordance with the tenets of the Declaration of Helsinki,^20^ and ethics approval was obtained from the Singhealth Centralised Institutional Review Board. Written informed consent was obtained from all participants. This study followed the Strengthening the Reporting of Observational Studies in Epidemiology (STROBE) reporting guideline for cohort studies.^21^

**Figure.**
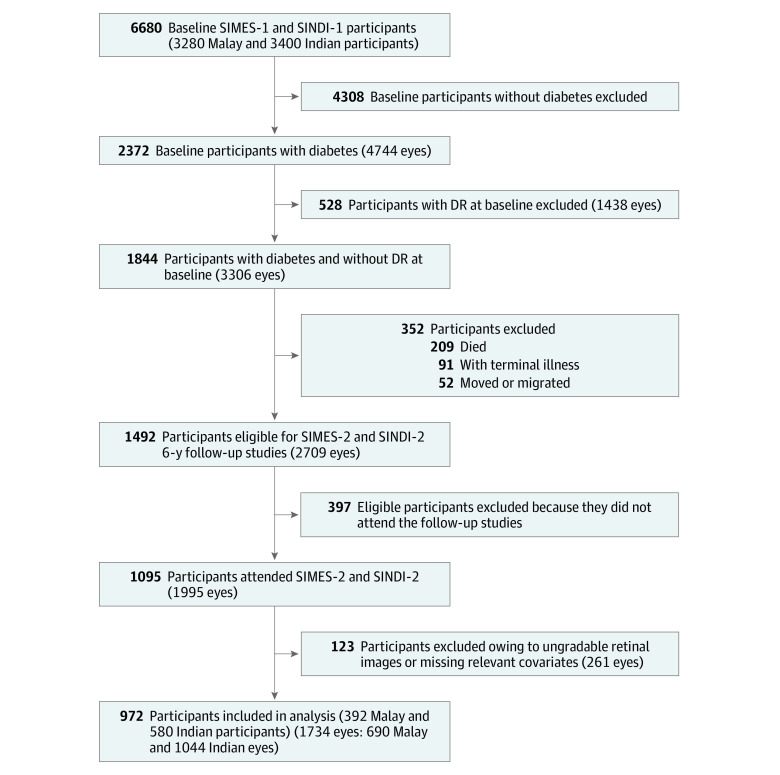
Flowchart for Inclusion and Exclusion of Study Participants DR indicates diabetic retinopathy; SIMES, Singapore Malay Eye Study; and SINDI, Singapore Indian Eye Study.

### Ophthalmic Examination

All participants underwent comprehensive ocular examinations according to a standard protocol at the baseline and follow-up examinations. After dilation, a digital slitlamp camera (Topcon model DC-1 with FD-21 flash attachment; Topcon) and a retroillumination camera (Nidek EAS-1000; Nidek) were used to photograph the lens for evaluation of lens status. Absence of crystalline lens (aphakia) and presence of intraocular lens implant (pseudophakia) were assessed and were collectively categorized as eyes with cataract surgery performed.

### Retinal Photography and DR Grading System

Two-field (optic disc–centered and fovea-centered) colored fundus photographs were taken according to the Early Treatment for Diabetic Retinopathy Study (ETDRS) standards.^22,23^ The photographs of both eyes were obtained using a digital retinal camera (Canon CR-DGi with a 10-D SLR back; Canon) after pupil dilation. Fundus photographs were graded by a single grader (for both baseline and follow-up visits) in a masked manner. The presence and severity of DR for each eye were graded according to the scale of the modified Airlie House classification system (ranging from 10 to 90, with 10 being no DR).^24^ The presence of any DR was defined as level 15 or higher.^25^ The severity of DR was further categorized into minimal nonproliferative DR (NPDR; levels 15-20), mild NPDR (level 35), moderate NPDR (levels 43-47), severe NPDR (level 53), and proliferative DR (level ≥60). In this present study, eyes with incidence of DR were defined as those with the presence of any DR (level ≥15) at 6-year follow-up with no DR at baseline; persons with DR were defined as those with incident DR in either eye. Progression of DR was defined as an increase in the severity of retinopathy by 1 step or more from baseline to the follow-up examination. Proliferative DR cases at baseline were excluded from DR progression–associated evaluation.

### Other Examinations and Definition

A comprehensive questionnaire-based interview was administered by trained interviewers to collect data on participants’ medical history and smoking status. Detailed physical examinations and blood tests were performed as described previously.^17,18,19^ In brief, blood pressure (BP) was measured using a digital automatic BP monitor (Dinamap model Pro Series DP110X-RW, 100V2; GE Medical Systems Information Technologies Inc) after 5 minutes of rest. Data on participants’ weight and height were also collected to calculate their body mass index (BMI; calculated as weight in kilograms divided by height in meters squared). A nonfasting venous blood sample (40 mL) was collected to measure participants’ hemoglobin A_1c_ (HbA_1c_) and random glucose levels.

Participants with diabetes were defined as those having a random glucose level of 199.8 mg/dL or more (to convert to millimoles per liter, multiply by 0.0555) and an HbA_1c_ level of 6.5% or more (to convert to proportion of total hemoglobin, multiply by 0.01), using any antidiabetic medication, having a self-reported history of diabetes, or having a history of a physician’s diagnosis of diabetes. Participants with hypertension were defined as those with a systolic BP of 140 mm Hg or more or a diastolic BP of 90 mm Hg or more, a self-reported history of hypertension, or a previous diagnosis of hypertension. Smokers were defined as those currently smoking (regardless of number of cigarettes).

### Statistical Analysis

Data analysis was performed from October 1 to 31, 2019, using statistical analytical software R, version 3.4.0 (R Project for Statistical Computing). For person-specific characteristics, an independent *t* test was used to compare continuous variables, and a χ^2^ test was used for categorical variables. Eye-specific data were used to assess the association between baseline cataract surgery status (exposure variable) and incident DR (outcome variable). Multivariable Poisson regression models with generalized estimating equations were used to account for correlations between both eyes. Model 1 was adjusted for age, sex, and race/ethnicity. Model 2 was further adjusted for other potential confounders, including baseline HbA_1c_ level, duration of diabetes, random glucose level, antidiabetic medication use, hypertension, BMI, and smoking status. All *P* values were from 2-sided tests and results were deemed statistically significant at *P* < .05.

## Results

A total of 972 participants (495 men; mean [SD] age, 58.7 [9.1] years) and 1734 eyes were included in the final analysis. The mean (SD) follow-up period was 6.3 (0.9) years. As shown in eTable 1 in the Supplement, at baseline, compared with the included participants, the excluded participants were older (mean [SD] age, 60.4 [10.1] years vs 58.7 [9.1] years; *P* = .002) and had a shorter mean (SD) duration of diabetes (4.3 [7.2] years vs 5.5 [6.8] years; *P* = .002), a higher mean (SD) systolic BP (147.6 [23.7] mm Hg vs 141.7 [19.8] mm Hg; *P* < .001), a higher mean (SD) diastolic BP (80.1 [11.0] mm Hg vs 78.7 [10.0] mm Hg; *P* = .02), a slightly lower random mean (SD) blood glucose level (160.2 [77.4] vs 172.8 [77.4] mg/dL; *P* = .002), and a lower rate of antidiabetic medication use (227 of 520 [43.7%] vs 582 of 972 [59.9%]; *P* < .001).

Table 1 shows the baseline characteristics of participants with diabetes with or without incident DR included in the final analysis. At baseline, compared with participants without incident DR, participants who developed DR were mostly male (118 of 202 [58.4%] vs 377 of 770 [49.0%]; *P* = .02), had higher rates of current smoking (35 of 202 [17.3%] vs 96 of 770 [12.5%]; *P* = .03) and antidiabetic medication use (134 of 202 [66.3%] vs 448 of 770 [58.2%]; *P* = .04), a higher mean (SD) HbA_1c_ level (8.7% [2.0%] vs 7.4% [1.4%]; *P* < .001), a higher random mean (SD) blood glucose level (212.4 [86.4] vs 163.8 [70.2] mg/dL; *P* < .001), a higher mean (SD) diastolic BP (80.4 [10.2] mm Hg vs 78.3 [9.9] mm Hg; *P* = .008), a longer mean (SD) duration of diabetes (6.6 [7.3] years vs 5.2 [6.6] years; *P* = .01), and a lower mean (SD) BMI (26.4 [4.4 vs 28.1 [4.8] ; *P* < .001). Among the 1734 study eyes, 163 study eyes had already undergone cataract surgery at baseline and 187 eyes (originally phakic at baseline) underwent cataract surgery at any time during the follow-up period. Of these 350 eyes, 77 (22.0%) developed DR. Among the 1384 eyes that never underwent cataract surgery, 195 (14.1%) developed DR.

**Table 1.** Baseline Characteristics of Participants With Diabetes With or Without Incident DR

Characteristic	Participants, No. (%)		*P* value^b^
	Without incident DR (n = 770; 1462 eyes)	With incident DR (n = 202; 272 eyes)^a^	
Age, mean (SD), y	59.0 (9.1)	57.7 (9.3)	.08
Male sex,	377 (49.0)	118 (58.4)	.02
Malay ethnicity	309 (40.1)	83 (41.1)	.87
Current smoking	96 (12.5)	35 (17.3)	.03
Diabetes duration, mean (SD), y	5.2 (6.6)	6.6 (7.3)	.01
Antidiabetic medication use	448 (58.2)	134 (66.3)	.04
Hypertension	572 (74.3)	149 (73.8)	.95
Blood pressure, mean (SD), mm Hg			
Systolic	141.2 (19.8)	143.8 (19.5)	.10
Diastolic	78.3 (9.9)	80.4 (10.2)	.008
Hemoglobin A_1c_, mean (SD), %	7.4 (1.4)	8.7 (2.0)	<.001
BMI, mean (SD)	28.1 (4.8)	26.4 (4.4)	<.001
Random blood glucose, mean (SD), mg/dL	163.8 (70.2)	212.4 (86.4)	<.001
Positive cataract surgery status^c^			
At baseline	132 (9.0)	31 (11.4)	.26
During 6-y follow-up	141 (9.6)	46 (16.9)	<.001
Follow-up time, mean (SD), y	6.3 (0.9)	6.4 (1.0)	.16

Abbreviations: BMI, body mass index (calculated as weight in kilograms divided by height in meters squared); DR, diabetic retinopathy.

SI conversion factors: To convert hemoglobin A_1c_ to proportion of total hemoglobin, multiply by 0.01; and glucose to millimoles per liter, multiply by 0.0555.

^a^ Incident DR in either eye.

^b^ Estimated based on χ^2^ or independent *t* test, where appropriate.

^c^ Based on eye-level data.

After excluding eyes that were phakic at baseline but underwent cataract surgery during the 6-year follow-up period, we observed that cataract surgery at baseline was associated with 2.07 times (95% CI, 1.34-3.20; *P* = .001) greater risk of developing DR, after adjusting for age, sex, race/ethnicity, baseline HbA_1c_ level, duration of diabetes, random glucose level, antidiabetic medication use, hypertension, BMI, and smoking status (Table 2). These significant associations were similarly observed in the subgroups of Malay participants (RR, 2.62; 95% CI, 1.42-4.84; *P* = .002) and Indian participants (RR, 1.92; 95% CI, 1.11-3.34; *P* = .02).

**Table 2.** Association Between Baseline Cataract Surgery and DR Incidence^a^

Model	Malay participants (n = 631 eyes)		Indian participants (n = 916 eyes)		Combined (n = 1547 eyes)	
	RR (95% CI)	*P* value	RR (95% CI)	*P* value	RR (95% CI)	*P* value
Model 1^b^	2.61 (1.42-4.81)	.002	2.07 (1.18-3.62)	.01	2.16 (1.43-3.26)	<.001
Model 2^c^	2.62 (1.42-4.84)	.002	1.92 (1.11-3.34)	.02	2.07 (1.34-3.20)	.001

Abbreviations: DR, diabetic retinopathy; RR, relative risk.

^a^ Baseline phakic eyes that subsequently underwent cataract surgery during the 6-year follow-up period were excluded for this analysis (n = 187).

^b^ Adjusted for baseline age, sex, and race/ethnicity in the combined group.

^c^ Adjusted for baseline age, sex, race/ethnicity, baseline hemoglobin A_1c_ level, diabetes duration, random glucose level, antidiabetic medication use, hypertension, body mass index, and smoking status.

In a further subgroup analysis, which only included originally phakic eyes at baseline (Table 3), we observed that eyes that underwent cataract surgery at any time during the follow-up period were also more likely to develop DR (RR, 1.64; 95% CI, 1.15-2.33; *P* = .006) compared with eyes that never underwent cataract surgery, after adjusting for the same set of covariates. This association was only significant for Indian participants (RR, 1.95; 95% CI, 1.28-2.96; *P* = .002) but not for Malay participants (RR, 1.53; 95% CI, 0.90-2.62; *P* = .12).

**Table 3.** Association Between Cataract Surgery (Performed During Follow-up Period) and DR Incidence, Among Baseline Phakic Eyes^a^

Model	Malay participants (n = 642 eyes)		Indian participants (n = 929 eyes)		Combined (n = 1571 eyes)	
	RR (95% CI)	*P* value	RR (95% CI)	*P* value	RR (95% CI)	*P* value
Model 1^b^	2.22 (1.32-3.74)	.003	2.18 (1.42-3.35)	<.001	2.13 (1.53-2.98)	<.001
Model 2^c^	1.53 (0.90-2.62)	.12	1.95 (1.28-2.96)	.002	1.64 (1.15-2.33)	.006

Abbreviations: DR, diabetic retinopathy; RR, relative risk.

^a^ In this subgroup analysis, eyes with positive cataract surgery status at baseline were excluded (n = 163). Among the remaining 1571 baseline phakic eyes, 1384 eyes remained phakic throughout the follow-up period, while 187 eyes subsequently underwent cataract surgery during the 6-year follow-up period. This subgroup analysis compares the risk of developing DR between these 2 groups of eyes.

^b^ Adjusted for baseline age, sex, and additionally for race/ethnicity in the combined group.

^c^ Adjusted for baseline age, sex, race/ethnicity, baseline hemoglobin A_1c_ level, diabetes duration, random glucose level, antidiabetic medication use, hypertension, body mass index, and smoking status.

Table 4 shows the association between any prior cataract surgery and DR incidence. After adjustment for baseline age, sex, and race/ethnicity, any prior cataract surgery was associated with increased risk of DR (RR, 2.08; 95% CI, 1.56-2.78; *P* < .001). After further adjustment for baseline HbA_1c_ level, duration of diabetes, random glucose level, antidiabetic medication use, hypertension, BMI, and smoking status, any prior cataract surgery was significantly associated with increased risk of DR (RR, 1.70; 95% CI, 1.26-2.30; *P* = .001). Similar associations were observed in subgroups of Malay participants (RR, 1.73; 95% CI, 1.11-2.69; *P* = .02) and Indian participants (RR, 1.93; 95% CI, 1.33-2.80; *P* < .001). In addition, the 77 incident DR cases with a positive cataract surgery history were largely mild or moderate DR types (eTable 2 in the Supplement).

**Table 4.** Association Between Any Prior Cataract Surgery and DR Incidence

Model	Malay participants (n = 690 eyes)		Indian participants (n = 1044 eyes)		Combined (n = 1734 eyes)	
	RR (95% CI)	*P* value	RR (95% CI)	*P* value	RR (95% CI)	*P* value
Model 1^a^	2.22 (1.45-3.41)	<.001	2.10 (1.44-3.06)	<.001	2.08 (1.56-2.78)	<.001
Model 2^b^	1.73 (1.11-2.69)	.02	1.93 (1.33-2.80)	<.001	1.70 (1.26-2.30)	.001

Abbreviations: DR, diabetic retinopathy; RR, relative risk.

^a^ Adjusted for baseline age, sex, and additionally for race/ethnicity in the combined group.

^b^ Adjusted for baseline age, sex, race/ethnicity, baseline hemoglobin A_1c_ level, diabetes duration, random glucose level, antidiabetic medication use, hypertension, body mass index, smoking status.

Furthermore, among the adjusted covariates, younger age, high HbA_1c_ level, longer diabetes duration, and lower BMI at baseline were also significantly associated with increased risk of incident DR. This association was consistently observed when evaluating the respective multivariable models (eTables 3, 4, and 5 in the Supplement). On the other hand, in addition to the risk of developing DR, we also separately evaluated whether cataract surgery was associated with a higher risk of DR progression. In a separate sample of 479 eyes (327 participants) with DR at baseline (but without proliferative DR), we found no significant association between previous cataract surgery and DR progression (RR, 0.99; 95% CI, 0.61-1.61; *P* = .98), after adjusting for the same set of covariates as our main analysis of DR incidence. Nevertheless, our current sample size for DR progression has a limited power of 47% to detect an RR of 1.5 and of 12% to detect an RR of 1.2 (assuming α level to be .05). Hence, future studies and meta-analysis are needed to further elucidate the association of cataract surgery with the risk of DR progression.

## Discussion

In this 6-year follow-up cohort study, we observed that individuals with diabetes from our Malay and Indian population-based cohorts had increased risk of developing DR after cataract surgery. This association was consistent in both racial/ethnic groups and remained significant even after accounting for relevant confounders. Nevertheless, this finding was observed mainly in incident cases of mild or moderate DR; thus, further validation is still required to confirm this observation.

Cataract surgery had previously been reported to exacerbate existing DR in patients with diabetes. However, there are limited studies that evaluated the association of cataract surgery with the incidence of DR. Previously, a case-control study using national health insurance data in Taiwan showed that history of cataract surgery was significantly associated with increased risk of developing NPDR.^26^ This association was also observed in incident DR cases 5 years after cataract surgery. Taken together with our 6-year follow-up finding (Table 2), this finding suggests that cataract surgery may indeed have a long-term association with the development of DR among individuals with diabetes. Furthermore, in our sensitivity analysis, after further excluding 3 aphakic eyes at baseline (which presumably had more invasive cataract surgery), the significant association between cataract surgery (only pseudophakic eyes) and incident DR still remained largely similar. Similarly, in a retrospective clinical study, Hong et al^11^ reported that patients with diabetes who underwent phacoemulsification had twice the rate of DR incidence (over 12 months) compared with patients who never underwent cataract surgery. These observations are supported by previous studies that reported that retinal blood flow and the blood-aqueous barrier may be disrupted during or after cataract surgery.^27,28^ In addition, increased levels of inflammatory biomarkers, such as interleukin 6, in the aqueous humors were also observed after cataract surgery.^29,30^ Taken together, it is plausible that these factors may collectively be associated with the development of DR after cataract surgery.

On the other hand, a prospective clinical study involving 205 diabetic eyes showed that eyes that underwent cataract surgery and eyes that did not undergo cataract surgery had similar DR incidence rates 6 months after surgery (18.3% vs 14.3%).^31^ This observation was inconsistent with our current findings. However, this study was limited by a small sample size and did not sufficiently adjust for relevant confounding risk factors that may have been associated with the outcome of interests. Our study’s strengths include the prospective design, large sample size, and long 6-year follow-up period between examinations.

### Limitations

This study has limitations. First, 397 of 1492 eligible individuals with diabetes (26.6%) did not return for follow-up. Considering that the excluded individuals were slightly older and had higher systolic and diastolic BP at baseline (compared with included participants), it is possible that the excluded individuals might have been more likely to develop DR. This possibility may potentially result in an underestimation of our current findings. Second, the presence of any DR was defined as a level 15 or more, which also translated to minimum detection of 1 microaneurysm or dot hemorrhage. Thus, in the case of minimal or mild NPDR, it remains possible that the true presence of these mild DR signs might have been obscured by mild lens opacities that might be present in eyes that did not undergo cataract surgery throughout the follow-up period. Such a scenario might have resulted in a false-negative classification for DR. Nevertheless, we expect this association to be minimal because eyes with ungradable retinal photographs were already excluded from the final analysis. Similarly, we cannot entirely rule out the possibility of detection bias because eyes that underwent cataract surgery were more likely to be diagnosed accurately for DR compared with eyes with lens opacities. Third, we were not able to include information on the exact time point that participants underwent cataract surgery because this information was either not completely or not accurately collected from participants owing to self-reporting error and recall bias. Fourth, because previous studies had reported that individuals with diabetes and cataract surgery complications (eg, retained lens material or intraocular lens reposition or exchange) had an increased risk of DR,^26,32,33^ the lack of detailed information on cataract surgery complications (among individuals who underwent cataract surgery) is also a limitation.

## Conclusions

Our population-based study demonstrated that prior cataract surgery was associated with a higher risk of developing DR among individuals with diabetes. Nevertheless, this risk was observed mainly in incident mild and moderate DR cases. Further studies are still warranted to validate this association. On further validation, it may be conceivable for patients with diabetes to be recommended for more frequent DR screenings after cataract surgery.

## Notes

Supplement. **eTable 1.** Baseline Characteristics of Included and Excluded Participants **eTable 2.** Stratified Tabulation of Incident DR Cases, by Baseline Cataract Surgery Status and DR Severity **eTable 3.** Multivariable Regression Model (Model 2 in Main Table 2) Showing the Associations Between Baseline Factors and DR Incidence **eTable 4.** Multivariable Regression Model (Model 2 in Main Table 3) Showing the Associations Between Baseline Factors and DR Incidence **eTable 5.** Multivariable Regression Model (Model 2 in Main Table 4) Showing the Associations Between Baseline Factors and DR Incidence
