# Supplementary material for: Association of Cataract Surgery With Risk of Diabetic Retinopathy Among Asian Participants in the Singapore Epidemiology of Eye Diseases Study
Source: JAMA Netw Open. 2020 Jun 16;3(6):e208035. doi: 10.1001/jamanetworkopen.2020.8035 (PMC7298610; doi:10.1001/jamanetworkopen.2020.8035)
Supplement: Supplement. — eTable 1. Baseline Characteristics of Included and Excluded Participants eTable 2. Stratified Tabulation of Incident DR Cases, by Baseline Cataract Surgery Status and DR Severity eTable 3. Multivariable Regression Model (Model 2 in Main Table 2) Showing the Associations Between Baseline Factors and DR Incidence eTable 4. Multivariable Regression Model (Model 2 in Main Table 3) Showing the Associations Between Baseline Factors and DR Incidence eTable 5. Multivariable Regression Model (Model 2 in Main Table 4) Showing the Associations Between Baseline Factors and DR Incidence [file jamanetwopen-3-e208035-s001.pdf]

Supplementary Online Content

Tham Y-C, Liu L, Rim TH, et al. Association of cataract surgery with risk of diabetic retinopathy among Asian participants in the Singapore Epidemiology of Eye Diseases Study. *JAMA Netw Open.* 2020;3(6):e208035. doi:10.1001/jamanetworkopen.2020.8035

eTable 1. Baseline Characteristics of Included and Excluded Participants
eTable 2. Stratified Tabulation of Incident DR Cases, by Baseline Cataract Surgery Status and DR Severity
eTable 3. Multivariable Regression Model (Model 2 in Main Table 2) Showing the Associations Between Baseline Factors and DR Incidence
eTable 4. Multivariable Regression Model (Model 2 in Main Table 3) Showing the Associations Between Baseline Factors and DR Incidence
eTable 5. Multivariable Regression Model (Model 2 in Main Table 4) Showing the Associations Between Baseline Factors and DR Incidence

This supplementary material has been provided by the authors to give readers additional information about their work.

© 2020 Tham Y-C et al. *JAMA Network Open.*

eTable 1. Baseline Characteristics of Included and Excluded Participants

	**Excluded (N=520)**	**Included (N=972)**	***P* Value***
Age, years	60.4 (10.1)	58.7 (9.1)	0.002
Gender (Male), %	241 (46.4)	495 (50.9)	0.103
Ethnicity (Malay),%	258 (49.6)	392 (40.3)	0.001
Current smoking, %	81 (15.6)	131 (13.5)	0.247
Diabetes duration, years	4.3 (7.2)	5.5 (6.8)	0.002
Anti-diabetic medication use, %	227 (43.7)	582 (59.9)	<0.001
Hypertension, %	403 (77.5)	721 (74.2)	0.175
Systolic blood pressure, mmHg	147.6 (23.7)	141.7 (19.8)	<0.001
Diastolic blood pressure, mmHg	80.1 (11.0)	78.7 (10.0)	0.023
HbA_1c, %	7.5 (1.6)	7.7 (1.6)	0.025
Body mass index, kg/m^2	27.5 (5.0)	27.7 (4.8)	0.503
Random blood glucose, mg/dL	160.2 (77.4)	172.8 (77.4)	0.002
Positive cataract surgery status at baseline**, %	78 (8.0)	163 (9.4)	0.247
Data presented as mean (standard deviation) or frequency (percentage), where appropriate.<br><br>*P value was estimated based on chi-square or independent t-test, where appropriate.<br><br>**Based on eye-level data.			

© 2020 Tham Y-C et al. *JAMA Network Open.*

eTable 2. Stratified Tabulation of Incident DR Cases, by Baseline Cataract Surgery Status and DR Severity

		Incident DR cases stratified by DR severity level*, number of eyes (%)				
**Eyes which had cataract surgery and developed DR**	**Number of eyes with incident DR**	Minimal NPDR (Level 15-20)	Mild NPDR (Level 35)	Moderate NPDR (Level 43-47)	Severe NPDR (Level 53)	PDR (>Level 60)
Positive cataract surgery status at baseline	31	17 (54.8%)	11 (35.5%)	2 (6.5%)	0 (0.0%)	1 (3.2%)
Positive cataract surgery status during 6-year follow-up	46	16 (34.8%)	14 (30.4%)	10 (21.7%)	2 (4.4%)	4 (8.7%)
**Overall**	**77**	**33 (42.8%)**	**25 (32.5%)**	**12 (15.6%)**	**2 (2.6%)**	**5 (6.5%)**

* According to the modified Airlie House classification system.

DR =diabetic retinopathy; NPDR = non-proliferative diabetic retinopathy; PDR = proliferative diabetic retinopathy.

© 2020 Tham Y-C et al. *JAMA Network Open.*

eTable 3. Multivariable Regression Model (Model 2 in Main Table 2) Showing the Associations Between Baseline Factors and DR Incidence

**Baseline Factors**	**RR (95%CI)**	***P* Value**
Age, year	0.96 (0.94-0.97)	<0.001
Gender (Male)	1.12 (0.76-1.65)	0.563
Ethnicity (Malay)	1.05 (0.78-1.43)	0.734
HbA_1c, %	1.32 (1.20-1.46)	<0.001
Diabetes duration, year	1.02 (0.99-1.04)	0.288
Random blood glucose, mmol/L	1.01 (0.97-1.05)	0.484
Anti-diabetes medication use	1.40 (1.00-1.95)	0.048
Hypertension status	1.31 (0.95-1.80)	0.100
Body mass index, kg/m^2	0.96 (0.92-0.99)	0.010
Current smoking	1.08 (0.68-1.71)	0.743
Baseline cataract surgery	2.07 (1.34-3.20)	0.001

RR = relative risk; CI = confidence interval; DR = diabetic retinopathy.

© 2020 Tham Y-C et al. *JAMA Network Open.*

eTable 4. Multivariable Regression Model (Model 2 in Main Table 3) Showing the Associations Between Baseline Factors and Incidence **of DR**

**Baseline Factors**	**RR (95%CI)**	***P* Value**
Age, year	0.97 (0.95-0.99)	0.001
Gender (Male)	1.08 (0.74-1.58)	0.691
Ethnicity (Malay)	1.21 (0.88-1.65)	0.238
HbA_1c, %	1.40 (1.27-1.54)	<0.001
Diabetes duration, year	1.04 (1.01-1.06)	0.002
Random blood glucose, mmol/L	0.99 (0.95-1.03)	0.684
Anti-diabetes medication use	1.20 (0.87-1.65)	0.262
Hypertension status	1.11 (0.82-1.50)	0.518
Body mass index, kg/m^2	0.97 (0.93-1.00)	0.033
Current smoking	1.11 (0.71-1.75)	0.636
Cataract surgery (performed during follow-up period)	1.64 (1.15-2.33)	0.006

RR = relative risk; CI = confidence interval; DR = diabetic retinopathy.

© 2020 Tham Y-C et al. *JAMA Network Open.*

eTable 5. Multivariable Regression Model (Model 2 in Main Table 4) Showing the Associations Between Baseline Factors and Incidence of DR.

**Baseline Factors**	**RR (95%CI)**	***P* Value**
Age, year	0.97 (0.95-0.99)	0.001
Gender (Male)	1.07 (0.74-1.54)	0.716
Ethnicity (Malay)	1.17 (0.87-1.57)	0.289
HbA_1c, %	1.35 (1.23-1.48)	<0.001
Diabetes duration, year	1.02 (1.00-1.05)	0.031
Random blood glucose, mmol/L	1.00 (0.96-1.04)	0.893
Anti-diabetes medication use	1.26 (0.93-1.70)	0.135
Hypertension status	1.18 (0.88-1.58)	0.269
Body mass index, kg/m^2	0.96 (0.93-0.99)	0.010
Current smoking	1.22 (0.81-1.85)	0.343
Any prior cataract surgery	1.70 (1.26-2.30)	0.001

RR = relative risk; CI = confidence interval; DR = diabetic retinopathy.

© 2020 Tham Y-C et al. *JAMA Network Open.*
